# MIDLIFE CREATIVITY AMONG COMPOSERS AND NOVELISTS

**DOI:** 10.1093/geroni/igac059.844

**Published:** 2022-12-20

**Authors:** Desmond O'Neill

**Affiliations:** Trinity College Dublin, Dublin, Dublin, Ireland

## Abstract

Although the study of midlife has increased in recent years, it still lags behind study of life at the extremes of age. As a key threshold stage in the lifespan, our understanding can be enhanced by exploration of novelists and composers whose most substantive work began to be produced in midlife. This presentation will draw off the works of midlife works of composers who also composed into later life - Giussepe Verdi, Richard Strauss and Johannes Brahms - from the concert proposed by the Indianapolis Symphony during GSA 2022, as well as reflecting on the works of Anita Brookner as representative of other novelists and poets whose work and careers came to prominence in midlife.

